# Hospital nurses’ knowledge about older patients in Turkey: a validation and comparison study

**DOI:** 10.1186/s12912-022-00882-6

**Published:** 2022-05-10

**Authors:** Deniz Harputlu, Sander Kerstens, Funda Özdemir, Jeroen Dikken

**Affiliations:** 1 Ankara University, Faculty of Nursing, Aktaş Mahallesi, Plevne Caddesi, No:5, 06080 Ankara, Altındağ Turkey; 2 Faculty of Health, Nutrition & Sport & Health Innovation Centre of Expertise, The Hague University of Applied Sciences, Johanna Westerdijkplein 75, 2521 EN The Hague, the Netherlands

**Keywords:** Cross-cultural validation, KOPQ, Knowledge, Nurses, Older patients, Turkey

## Abstract

**Background:**

In Turkey, nursing care in hospitals has gradually included more older patients, resulting in a need for knowledgeable geriatric nurses. It is unknown, however, whether the nursing workforce is ready for this increase. Therefore, the aim of this study is to validate the Knowledge about Older Patients Quiz (KOPQ) in the Turkish language and culture, to describe Turkish hospital nurses’ knowledge about older patients, and to compare levels of knowledge between Turkish and Dutch hospital nurses.

**Method:**

First, the KOPQ was translated, resulting in the KOPQ-TR. Then, content validity was assessed by 10 geriatric experts using the Lynn method, a pilot test among 10 nurses was conducted, and a Rasch analysis was performed using data from 135 nurses working in two Turkish hospitals. Finally, a comparison between Turkish and Dutch nurses’ levels of knowledge was performed.

**Results:**

The results of the qualitative validation (i.e., content validity by experts and nurses), model fit, item reliability and the item separation index of the KOPQ-TR proved excellent, indicating good content and construct validity. However, the Person Separation Index and Person Reliability of the Rash analysis did not meet the criteria for adequate scale and psychometric validation. The levels of knowledge among Turkish nurses were significantly lower than those of Dutch nurses.

**Conclusions:**

The KOPQ-TR is promising for use in Turkey, although psychometric validation should be repeated using a better targeted sample with a larger ability variance to adequately assess the Person Separation Index and Person Reliability. Currently, education regarding care for older patients is not sufficiently represented in Turkish nursing curricula. However, the need to do so is evident, as the results demonstrate that knowledge deficits and an increase in older patients admitted to the hospital will eventually occur. International comparison and cooperation provides an opportunity to learn from other countries that currently face the challenge of an aging (hospital) population.

**Supplementary Information:**

The online version contains supplementary material available at 10.1186/s12912-022-00882-6.

## Introduction

The world’s population is aging, and the prevalence of people aged 60 years and older is expected increase to approximately 22% by 2050. Countries face challenges in adapting their health care systems to this demographic shift [1]. In Turkey, the proportion of older people (defined as the population at 65 years of age and over) was 8.7% in 2018 and is expected to be 10.2% in 2023 and 22.6% in 2060 [2]. This demographic shift in Turkey also results in nurses who will increasingly encounter older patients [3]. Older patients are more likely to experience multiple chronic health conditions and often need additional support for activities of daily living [4]. This complex and vulnerable group of patients requires nurses to develop specific gerontological skills and possess excellent knowledge and attitudes with respect to caring for these patients [5, 6].

Several studies have previously assessed the knowledge and attitude of nurses [6–11], showing that negative attitudes were directed at the high care demand (e.g., time consumption, a burden) of older patients, their characteristics related to old age and nurses’ approaches in providing care (e.g., patient-centered and shared decision-making). Hanson (2014) found that a lack of knowledge of the gerontological and aging process of older people can negatively affect nurses’ attitudes towards older patients [12], emphasizing the importance of measuring nurses’ knowledge about older patients. In Turkey, the literature on nurses’ knowledge and attitudes towards caring for older patients is limited. One study, conducted by Adıbelli and Kılıç (2013), determined nurses’ attitudes towards older patient care and the difficulties they experience in Turkey [13]. The results of this descriptive study showed that nurses’ overall attitude towards older people was positive; however, insufficient knowledge, skills and experience with older patient care was found to be one of the difficulties nurses experienced [13]. Another cross-sectional study performed by Birimoğlu Okuyan, Bilgili and Mutlu (2020) investigated Turkish nursing students’ intention to work as a geriatric nurse and the factors influencing those intentions [14]. The study demonstrated that students avoid careers in geriatric nursing due to the lack of knowledge and skills and negative experiences during internships in clinical practice [15]. Gaining more insight into this ‘lack of knowledge’ about older patients among Turkish nurses could provide a clear direction for the education and training of nurses in the hospital setting.

Measuring knowledge among nurses is a difficult task due to the lack of valid measurement tools that solely assess nurses’ knowledge about older patients. Often, the knowledge domain has been incorporated as part of attitude in the results presented [7,8,10), making it difficult to interpret and analyze results on knowledge about older patients solely. Therefore, a measurement tool must solely assess knowledge, without the related and often overlapping constructs, such as attitudes and prejudices. To fill this gap in the literature, Dikken et al. developed a valid and reliable instrument to assess solely the levels of knowledge about older patients among nursing students and hospital staff: the Knowledge about Older Patients Quiz (KOPQ) [15, 16]. The KOPQ has already been validated in the United States of America [17]. However, before this instrument can be used in Turkey, cross-cultural validation is necessary. Sousa & Rojjanasrirat (2010) support the need to cross-culturally validate research instruments or scales to enable researchers to conduct cross-cultural research and to provide access to valid measurement tools to different countries [18]. To help researchers in this required validation process, they described a 7-step guideline to translate, adapt and cross-culturally validate measurement tools [18]. Due to the worldwide aging population and the need to assess the knowledge of nurses about older patients, it is important to have a cross-culturally validated measurement tool to enable researchers to compare countries (with each other) to explore strengths and weaknesses between countries and to learn from each other.

The aims of this study are to validate the KOPQ in the Turkish language and culture, to describe Turkish hospital nurses’ knowledge about older patients, and to compare the levels of knowledge between Turkish and Dutch hospital nurses.

## Methods

To perform cross-cultural validation of the Knowledge about Older patients Quiz - United States of America (KOPQ-US) for Turkish (KOPQ-TR), the steps as described by Sousa et al. (2010) were followed using a (multicenter) cross-sectional design. Turkish nurses’ knowledge level were interpreted. Then, these results were compared with previously published results obtained from Dutch nurses. Study procedures were reviewed and approved by the Ankara University Ethical Committee on June 18, 2019—approval number 227. Furthermore, the two participating hospitals provided formal approval for this study.

### Cross-cultural validation of the knowledge about older patients quiz

#### The knowledge about older patients quiz

The level of knowledge was measured by the KOPQ [15, 16]. The KOPQ contains 30 dichotomous items (true/false) measuring general knowledge about older hospitalized patients across six themes: normal aging, geriatric conditions, signaling problems with old age, interventions, family interventions and vulnerable patients versus older patients [15]. Every correct answer is assigned 1 point, and every incorrect answer is assigned 0 points [16]. The KOPQ was developed and validated for the Netherlands and exhibited adequate face validity, good readability [15], a good scale content validity index/average (S-CVI/ave. = .91) [16].

Psychometric validity of the KOPQ was previously assessed using item response theory [16] to determine the discrimination and difficulty parameters. Most items on the KOPQ had moderate to high discrimination values (indicating the extent to which the item is good for discriminating between knowledgeable and less-knowledgeable respondents [19]). The range at which the KOPQ retrieves information about the knowledge level of participants (difficulty) is β − 10.2 to 0.7, indicating that most items are easy to answer even if levels of knowledge are low [16]. Finally, the reliability for all knowledge items was considered good (internal consistency by Kuder-Richardson Formula 20 = .70) [16]. The KOPQ was proven to be cross-culturally valid for use in the United States of America, as full configural invariance and full metric invariance were established across countries [17]. This translated version of the KOPQ was used for translation into the Turkish language.

#### Translation of the knowledge about older patients quiz

First, the validated American-English version of the KOPQ was forward-translated into the Turkish language by three independent translators whose native language was Turkish (step 1, Sousa, 2010 [18]). All the translators were bilingual (English and Turkish). The first translator was knowledgeable about health care terminology and nursing care. The second and third translators were not knowledgeable about medical terminology. The three forward-translated versions of the KOPQ were initially compared by the researchers DH and FÖ (step 2, Sousa, 2010 [18]). These researchers are also bilingual. Consensus was achieved between researchers on all KOPQ items, resulting in an initial Turkish version of the KOPQ. Second, the initial Turkish version of KOPQ was translated back into English by one other independent translator (step 3, Sousa, 2010 [18]). This translator’s native language was English, and they were completely blind to the original version of KOPQ-US and not knowledgeable about medical terminology. This translator produced back-translated versions of the instrument. Third, the instructions, items and response format of the back-translation were compared with the instructions, items and response format of the original KOPQ-US by the researchers (DH and FÖ). The comparisons assessed the format, wording, and grammatical structure of the sentences; the similarity in meaning; and the relevance (step 4, Sousa, 2010 [18]). This process yielded in the Knowledge about Older Patients Quiz – Turkey (KOPQ-TR).

#### Initial validation of the knowledge about older patients quiz – Turkey

##### Setting and subjects

To assess the content validity of the KOPQ-TR (step 5, Sousa, 2010 [18]), the initial KOPQ-TR and the original KOPQ-US were sent to 10 faculty members who were experts in nursing and older person care via e-mail. Experts were asked to evaluate and score each item of the KOPQ-TR according to five evaluation criteria:

- Do the items in the translated version align with their meaning in the original scale?
- Is the comprehensibility and meaning equivalence achieved in Turkish culture for the items of the translation version?
- Do the items represent the property to be measured?
- Is the item clearly and simply expressed?
- Is the item understandable to the target audience?

With these criteria in mind, experts were asked to score each item on a 4-point Likert scale (1 = not suitable, 2 = a little suitable, 3 = quite suitable, 4 = extremely suitable). In addition, experts were asked to write suggestions for the items they gave 1 and 2 points.

##### Analysis

First, to assess the content validity, expert scores were dichotomized by summarizing scores 1 and 2 (not suitable) and scores 3 and 4 (suitable). The item-content validity index (I-CVI) was the result of the scores on one item divided by the number of experts. For an individual item to be considered suitable, the literature suggests that its I-CVI value should be greater than 0.78 [20, 21]. With 10 experts, the threshold used in this study was set to I-CVI = 0.80 or greater. For complete scale validation, all I-CVI values were averaged to calculate a Scale-Content Validity Index (S-CVIave), for which a value greater than 0.90 was considered excellent [20, 21]. Data were analyzed using SPSS version 22.0 [22].

##### Pilot testing of the Turkish version of the KOPQ

For the pilot test of the KOPQ-TR (step 5, Sousa, 2010 [18]), the comprehensibility and implementation process of the developed scale were evaluated by testing the scale with 10 nurses who were not included in the sample of psychometric testing. The researchers (DH and FÖ) asked nurses to read the items and provide direct feedback regarding whether the items were not understandable or were unclear.

#### Psychometric testing of the knowledge about older patients quiz-TR

##### Setting and subjects

In this study (step 7, Sousa, 2010 [18]), data were collected from nurses over a four-month period in Turkey. Nurses working in two university hospitals located in the capital city of Turkey were recruited. Clinical wards with older patients admitted regularly were included. The data were collected by researchers in Turkey (DH and FÖ) by going to the clinics and administering questionnaires. In every clinic, nurses were invited to participate by researchers with face-to-face meetings. Nurses were included only after informed consent was obtained. Hard copies of the KOPQ-TR were completed by nurses with pens. Included participants had to have a minimal age of 18 years old; red, wrote and understood Turkish; agreed to participate in the research; and work as a nurse in an adult unit of one of the participating hospitals.

##### Analysis

For assessment of the psychometric properties of the KOPQ-TR, a Rasch analysis was performed using JMetrik [23]. Rasch analysis is a form of item response theory and can be used for analyses of the psychometric properties of composite measures such as educational tests and health scales, which are considered to capture a unidimensional construct [24]. The Rasch model provides measurement that is not dependent on the distribution of the persons, given that the data fit the model [25], which implies that no assumptions about the person distribution have to be made. First, listwise deletion was used with participants having missing values on KOPQ-TR items. Second, unidimensionality, which is required by the Rasch model, is assessed using item ‘fit statistics’ and testing the assumption of local independence. Items with an infit weighted mean square (WMS) value of 0.7–1.3 are considered acceptable [26]. Values below 0.7 may indicate redundancy, and values over 1.3 indicate an unacceptable level of “noise” in the responses. Outfit values (UMS) are also considered, although they are more susceptible to outliers. Moreover, it was expected to extract no principal components for unidimensionality to hold [27]. Chou and Wang (2010) found that the longer the test and the smaller the sample, the larger the maximum of the first eigenvalue will be [28]. Because their results demonstrated that a fixed cutoff point for the first eigenvalue (e.g., 1.5) is infeasible for the determination of dimensionality, we interpreted a first eigenvalue score between 1.5 and < 3.5 in combination with the first contrast of residuals (i.e., the second dimension) of < 2.0 eigenvalues [29] as acceptable due to the small sample and relatively large test length in this study.

The Pearson separation index (PSI) and reliability (PR) are used to classify people [30]. Low person separation (< 2, person reliability < 0.8) with a relevant person sample implies that the instrument may not be sensitive enough to distinguish between high and low performers [31]. Person reliability depends chiefly on 1) sample ability variance, 2) length of test, 3) number of categories per item, and 4) sample-item targeting [32]. Item separation is used to verify the item hierarchy. Low item separation (< 3 = high, medium, low item difficulties, item reliability < 0.9) implies that the person sample is not large enough to confirm the item difficulty hierarchy (=construct validity) of the instrument. Item reliability depends chiefly on the 1) item difficulty variance and 2) sample size [32].

### Assessment of Turkish nurses’ knowledge and related variables

The same sample used to validate the KOPQ-TR was used to interpret Turkish nurses’ knowledge levels, and the relation between sociodemographic data and KOPQ-TR mean scores was examined via t-tests, Pearson correlations and one-way ANOVA. Moreover, as knowledge is closely related to attitude constructs such as opinions and preferences regarding working with older patients [33, 34], three additional questions were formulated by the Dutch research group [35], which we replicated for this study. First, nurses were asked which patient age category they preferred to work with (age 0–18, 19–69, 70+). Second, nurses were asked how they felt about the increase in older patients in the hospital (indicated on a scale from 1, no problem et al., to 10, a major problem). Finally, nurses were asked whether they find it difficult to care for older patients (indicated on a scale from 1, very easy to 10, very difficult). Additional demographic information was also collected to assess potentially contributing factors associated with nurses’ attitudes towards gerontology care, such as age, level of education and years of working experiences and type of ward [36].

### Comparison of Turkish and Dutch nurses’ knowledge

Turkish nurses’ knowledge results were compared with previously published data of Dutch nurses [35]. Differences between groups were tested with independent sample *t*-tests. Data were analyzed using SPSS version 22.0 [22].

## Results

### Results of cross-cultural validation of the knowledge about older patients quiz

#### Initial validation of the knowledge about older patients quiz-TR

For content and language validity, opinions of 10 experts were received, and the content validity index of the items (I-CVI) and total test (S-CVI) were calculated. For each item, the I-CVI was found to be between 0.80–1.00, and the S-CVI was 0.98 (see online Appendix 1). The pilot test results demonstrated that all nurses agreed that the items were clear.

#### Psychometric testing of the knowledge about older patients quiz-TR

##### Survey response and sample characteristics

In the participating sample, two nurses (1.5%) had missing values in the KOPQ items and were subsequently excluded. The sociodemographic characteristics of the 135 nurses with no missing data on the KOPQ are presented in Table 1.

**Table 1:** Characteristics of participants with no missing KOPQ-TR values (*n* = 135)

Characteristics of Participants	n (%), mean ± SD
Gender	
Female	123 (91.1)
Male	12 (8.9)
Age	34.5 ± 8.27
Hours working per week as a nurse	40.2 ± 2.2
Highest qualification	
Nursing high school	8 (5.9)
Nursing vocational school	14 (10.4)
Bachelor in nursing degree	104 (77.0)
Master in nursing degree	9 (6.7)
Distribution of nurses according to the ward they work in	
Surgical wards	49 (36.3)
Internal wards	86 (63.7)

##### Psychometric validity of the KOPQ-TR

Only items 14 and 23 demonstrated outfit values of 0.60 and 0.66, respectively. All other items demonstrated values between 0.7–1.3 and were considered acceptable (Table 2). The first eigenvalue of KOPQ-TR scored 3.07, followed by the second eigenvalue of 1.97; therefore, KOPQ-TR is considered unidimensional. The Pearson separation index and reliability of the KOPQ-TR did not meet the criteria for adequate scales (PSI = 0.282, PR = 0.073), but the item separation index and reliability did (ISI = 6.177, IR = 0.975).

**Table 2:** Difficulty structure and fit statistics for the 30-Item Knowledge about Older Patient Quiz - TR

Item description	Difficulty ***(std. error)***	Infit	Outfit
1. Forgetfulness, concentration issues, and indecisiveness are parts of aging rather than indicators of depression.	1.59 *(0.19)*	0.99	0.98
2. Unexpected urinary incontinence in an older person may indicate that the person is suffering from a urinary tract infection.	0.39 *(0.18)*	1.06	1.09
3. Patients with a cognitive disorder, such as dementia, are at greater risk for delirium.	−1.19 *(0.26)*	1.00	0.05
4. Malnutrition can have negative effects on thinking and observation skills.	−2.30 *(0.40)*	0.90	0.71
5. In general, older people are more sensitive to medication because their kidney and liver functions are declining.	−1.77 *(0.32)*	0.94	0.83
6. Meeting with families during patient assessment is required only for persons suffering from dementia.	−1.40 *(0.28)*	0.96	0.99
7. For older people, bed rest is important to enhance recovery.	1.00 *(0.18)*	1.02	1.03
8. Patients rarely remember that they were anxious and/or restless during delirium.	2.19 *(0.22)*	0.95	0.96
9. Older people need less fluid because they exercise less.	−0.50 *(0.21)*	0.90	0.85
10. Asking patients whether they have fallen in the past 6 months is a good way of assessing risk of falling.	0.17 *(0.18)*	1.04	1.05
11. Pressure that cuts off the blood supply to tissue for two hours may result in pressure ulcers.	−2.00 *(0.36)*	0.91	0.74
12. Depression is recognized in older people less frequently than it is in younger people.	−0.34 *(0.20)*	1.00	0.98
13. Lowering the frequency of a medication is an effective intervention to achieve (medication) adherence by patients.	1.28 *(0.18)*	1.02	1.03
14. Incontinent patients must have their soiled clothing changed but do not need to be placed on the toilet afterwards.	−2.71 *(0.49)*	0.89	0.60
15. It is good to have older people drink more often, because they have a reduced thirst sensation.	−1.19 *(0.26)*	0.98	1.02
16. In the case of delirium, bright lighting should be used to illuminate all of the corners of the room.	−0.59 *(0.21)*	0.98	1.03
17. Medication may cause geriatric problems such as memory deficits, incontinence, falling, and depression.	−0.38 *(0.20)*	0.92	0.85
18. Overburdening of family caregivers may lead to abuse of the person for whom they are providing care.	−0.94 *(0.24)*	0.95	0.86
19. It is good to provide extensive instruction about how to complete tasks to patients with apraxia.	2.49 *(0.24)*	1.01	1.09
20. When speaking to hearing-impaired older patients, it is best to speak at normal volume.	2.28 *(0.22)*	0.96	0.92
21. An older person with a BMI of > 25 cannot be undernourished.	0.58 *(0.18)*	0.92	0.91
22. In the case of difficulty swallowing, all medicines must be ground to ensure that patients ingest them.	1.35 (*0.18)*	1.00	1.00
23. In the case of depression, memory problems may occur.	−1.57 *(0.30)*	0.88	0.66
24. Most family caregivers do not need additional support from homecare services.	−1.26 *(0.27)*	0.96	0.90
25. As a nurse, you have to speak clearly into the ear of hearing-impaired older patients.	3.04 *(0.28)*	0.93	0.98
26. Pain medication should be administered to older people as little as possible, due to the possibility of addiction.	1.59 *(0.19)*	1.01	1.03
27. We identify pressure ulcers only if blister formation or abrasions have occurred.	−2.71 *(0.49)*	0.93	0.99
28. In the case of delirium, activities should be spread out evenly over the day.	−0.73 *(0.22)*	1.03	1.04
29. The risk of falling is higher for people in the hospital setting than in those who are living at home.	0.51 *(0.18)*	1.03	1.03
30. Stress incontinence may occur in patients who are not capable of opening their own trousers.	3.12 *(0.29)*	0.97	1.16

**Fig. 1:**
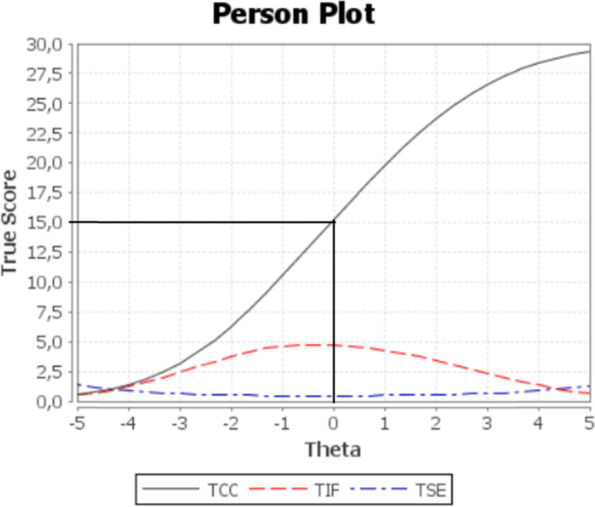
Person plot for the KOPQ-TR

The item difficulty hierarchy of KOPQ-TR items varied between − 2.71 (Item 14) and 3.12 (Item 30); see Table 2. Fig. 1 presents the test characteristic curve in the black line (i.e., the functional relation between the true score and the ability scale), test information function as the red line (i.e., the “statistical information” in the data corresponding to each score or measure on the complete test) and the standard error of the Rasch measure as the blue line. It is shown that information is retrieved over a wide range of ability levels (± − 3–3), meaning that items are well distributed across the Rasch continuum.

### Turkish nurses’ knowledge about older patients

Turkish nurses’ KOPQ-TR mean score was 18.81 ± 2.14 (min: 14-max: 24). Table 3 shows the relations between nurses’ sociodemographic characteristics and KOPQ-TR mean scores. There was no relation between the KOPQ-TR mean score and nurses’ age, working hours per week as a nurse, gender, or education level (*p* > 0.05).

**Table 3:** Turkish nurses’ knowledge and related variables

	KOPQ -TR mean score ± SD	Test statistics and *p* value
Age	–	r* = −0.107
		*p* = 0.221
Hours working per week as a nurse	–	r* = 0.20
		*p* = 0.818
Gender		
Female	18.87 ± 2.16	t* = 1.093
Male	18.16 ± 1.89	*p* = 0.656
Education Level		
Nursing high school	17.87 ± 1.95	
Nursing vocational school	18.00 ± 2.63	F* = 1.427
Bachelor in nursing degree	18.99 ± 2.02	*p* = 0.238
Master in nursing degree	18.88 ± 2.75	
KOPQ-TR mean score ± SD	18.81 ± 2.14	

*r = pearson correlation, t = t test, F = one way anova

### Comparison of Turkish and Dutch nurses’ knowledge

**Fig. 2:**
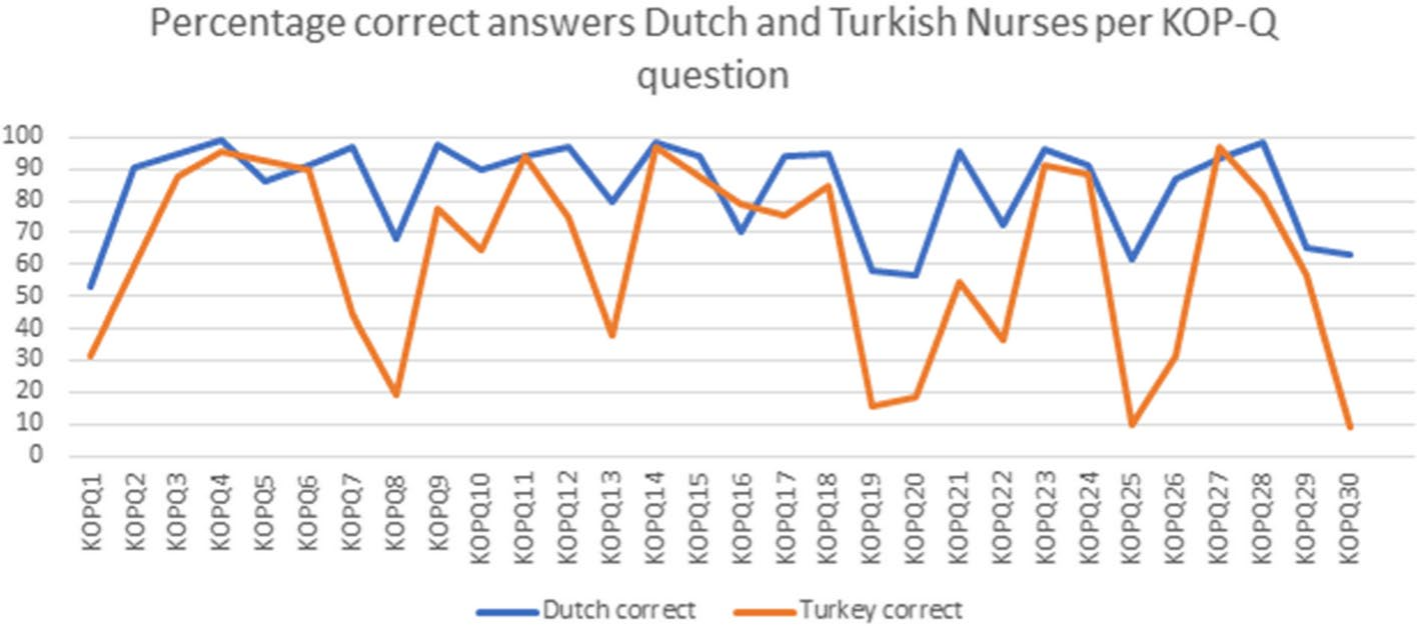
Percentage correct answers Dutch and Turkish nurses per KOPQ question trend line

Figure 2 shows for each question of the KOPQ the percentage of correct answers per country (see numerical data online appendix 2). Overall, Turkish nurses scored lower than Dutch nurses on most KOPQ items. There is a similarity between both countries on which items are considered difficult and easy, demonstrated by a similar trend line (i.e., difficult questions score lower in both Turkey and the Netherlands and vice versa for easier questions). The difference in scores between the two countries increases for questions that are perceived as difficult.

#### Knowledge in relation to opinions and preferences

In Table 4, Turkish nurses’ opinions and preferences are plotted against Dutch data published by Derks et al., 2021. Of the Turkish nurses participating in the study, only 1.5% answered the question “which target group would you prefer to work with” positively regarding care for older patients. Almost all nurses (94.8%) preferred to care for middle-aged patients, and only 3.7% of nurses preferred to care for children, which is unsurprising, as nurses working in pediatric wards were excluded from participation in this study. These percentages were not very different from those in the Netherlands: 12.6% of nurses preferred to work with older people, and 77.6% preferred to work with middle-aged patients (ΔM = −.049; *p* = .031). Additionally, worries about an increase in older patients in the hospital did not significantly differ between the two countries (ΔM = .415; *p* = .132). However, Turkish nurses did have significantly lower levels of knowledge than Dutch nurses (ΔM = − 6.326; *p* < .000). Moreover, Turkish nurses find caring for older patients to be difficult (M = 7.30; SD = 2.30), whereas Dutch nurses believe that caring for older patients is easy (M = 3.54; SD = 2.04); this difference was significant (ΔM = 3.753; *p* < .000).

**Table 4:** Comparison of knowledge in relation with opinions and preferences between Turkish and Dutch nurses

	Turkey (*n* = 135) n (%), mean ± SD	Netherlands^*^ (*n* = 1667) n (%), mean ± SD	Mean diff.	Sig. (95% CI)
Which target group would you prefer to work with?			−0.049	.031 (−.094 – -.004)
0–18	5 (3.7)	164 (9.8)		
19–69	128 (94.8)	1294 (77.7)		
70+	2 (1.5)	209 (12.5)		
What do you think of an increase in the number of older people? (Scale 1–10)^a^	5.98 ± 3.11	5.56 ± 2.41	.415	.132 (−.127–.957)
Do you find it difficult to take care for older patients? (Scale 1–10)^b^	7.30 ± 2.30	3.54 ± 2.04	3.753	<.000 (3.392–4.115)
Mean KOP-Q score	18.81 ± 2.14	25.14 ± 2.37	−6.326	<.000 (−6.739 – −5.914)

^*^Turkish data is compared using independent t-test with Dutch data from Derks at al., 2021 [33]

^a^Nurses were asked how they feel about the increase of older patients in the hospital (indicated on a scale from 1 (no problem et al) to 10 (a big problem))

^b^Nurses were asked whether they find it difficult to care for older patients (indicated on a scale from 0 (very easy) to 10 (very difficult))

## Discussion

This study assessed the (initial) validity of the KOPQ-TR and interpreted the results of Turkish hospital nurses participating in the research in comparison with Dutch hospital nurses. For cross-cultural validation of the KOPQ, all steps as described by Sousa, 2010 [18] were correctly executed, and the results indicate that the KOPQ-TR is a promising instrument to assess nurses’ knowledge about older patients in Turkey. The average knowledge level of Turkish nurses was significantly lower than the average of Dutch nurses, and they found that caring for older people was more difficult.

Although all steps for translation and initial validation by Sousa, 2010 [18] were correctly executed and the results of the qualitative validation (i.e., content validity by experts and nurses) looked promising, the Person Separation Index and Person Reliability of the KOPQ-TR did not meet the criteria for adequate scales [31]. Because only hospital nurses participated in the study, the sample proved too homogeneous in ability variance. We believe the psychometric assessment of the KOPQ-TR should be repeated with a better targeted sample having a larger ability variance (e.g., first year students, final year students, geriatric specialists) to ascertain analysis are executed on data having sufficient ability variance for assessment of Person Reliability and Separation Index adequately [32]. Replication using a different sample is encouraged, especially because the model fit, item reliability and the item separation index proved excellent, indicating good item difficulty variance (i.e., construct validity) for the KOPQ-TR. Moreover, by validating the KOPQ-TR using known group levels of knowledge(e.g., first-year students, final-year students, geriatric specialists), norm reference groups can be formulated that are useful for individual test takers to interpret their own test results [16].

The mean KOPQ score among Turkish nurses participating in this study was significantly lower than that of nurses working in the Netherlands. One of the reasons for this difference might be the lack of theoretical and practical training on geriatrics and geriatric nursing in the nursing education curriculum in Turkey. A study conducted by Adıbelli and Kılıç with the participation of 282 nurses in Turkey stated that one of the difficulties faced by nurses in older person care is insufficient knowledge, practice and experience [13]. Another study published on this subject in Turkey was conducted as a descriptive study with the participation of 227 nurses. One of the results of this study is that only 33% of the nurses received training in geriatrics [37]. One of the studies on geriatric nursing with nursing students in Turkey was carried out by Bakan et al. (2018) with the participation of 166 nursing students [38]. The results of this study showed that nursing students have positive attitudes towards older individuals [38]. However, according to the results of the cross-sectional study conducted by Birimoğlu Okuyan et al. (2020), which examined the factors affecting the career choice of nursing students, with the participation of 688 students, 63% of the participants stated that they did not take a separate geriatric nursing course, and 69% stated that they did not have experience in giving care to older patients [14]. In addition, all available studies suggest that separate geriatric nursing courses should be added to the nursing education curriculum and postgraduate education programs in Turkey.

Another reason for the difference in the KOPQ scores between nurses working in Turkey and nurses in the Netherlands might be due to the difference in the prevalence of older individuals in Turkey and the Netherlands. Turkey is a country with a younger population than European countries, but the ratio of the older population/total population is increasing gradually [39]. Despite this increasing trend, the proportion of the older population is lower than that in the Netherlands. According to Eurostat data, the population aged 65+ constitutes 9.1 of the total population in Turkey and 19.5 in the Netherlands [40]. This situation may lead to a difference between the two countries in the urgency for educators to plan and educate health care professionals about older patients. An interesting finding in this study showed that Turkish nurses find and experience it more difficult to work with older patients than Dutch nurses. In this finding lies an opportunity for education since Fox and Miner (1999) showed that health care professionals are motivated to learn when they experience a gap between what is and what needs to be [41]. In other words, Turkish nurses experience a greater need to learn and thus will be more eager to participate in an educational course.

A few questions demonstrated a large difference between countries in correct answer rate (İtem no; 7, 8, 13, 21, 25, 26, 30). A possible explanation for the large differences in questions regarding the themes bed rest, delirium, medication, caregivers, approach to the older person with hearing problems, and incontinence care are the national campaigns in the Netherlands focusing on implementing safety management systems (SMSs), where care for frail older patients is an important focus point of the program [42]. Baines et al. (2015) calculated that after the implementation of the SMS program in Dutch hospitals, the number of adverse events decreased by 30% [42]. Part of this system is also to make nurses aware of the risks of working with different high-risk patients and thus also with frail older patients. The World Health Organization (WHO) also advises programs such as those conducted in the Netherlands to support the education of health care professionals [43].

Some strengths and limitations of this study should be addressed. First, the number of participants to item ratio for the psychometric analysis was rather small and too homogeneous in ability variance, which could have influenced the Person Separation Index and Person Reliability of the KOPQ-TR [31], and replication with a larger sample with a diverse knowledge ability level is needed. However, other validity and reliability measures (both qualitative and quantitative) are promising. A strength of this study was the comparison of the knowledge of nurses about older patients among different countries and cultures. In doing so, we experienced that collaboration between researchers in the cross-cultural validation process (e.g., discussion of methods and results, sharing data) supported dialog and learning from each other’s context and cultural challenges, which provided interesting food for thoughts on how nurse researchers, educators and policy-makers could work together to enhance education in their respected countries. Therefore, when cross-cultural validation is needed, we recommend that researchers collaborate with the authors of the original scale, enhance knowledge exchange, facilitate learning from and with each other and create a network of cooperating countries that can share experiences, thereby increasing the quality of instruments and possible interventions used.

## Conclusion

This study identified that the KOPQ-TR can be considered initially valid for assessing nurses’ knowledge in Turkey. Translation, content validity and construct validity measures were excellent, but replication with a better targeted sample having a larger ability variance is still recommended as Person statistics were poor. Moreover, overall knowledge ability levels between Turkey and the Netherlands differed substantially. Turkish nurses demonstrated lower knowledge levels, and this difference in scores between both countries increased for questions that were perceived as difficult. In addition, Turkish nurses acknowledge that they perceive care for older patients as difficult which confirms the need and urgency to prioritize educational and quality improvement programs. Although the percentage of older patients in Turkish hospitals is increasing gradually, there is an opportunity to learn from other countries that currently face the challenge of an aging (hospital) population. By addressing shortcomings in nurses’ knowledge about older patients in Turkey at a national level, including all relevant stakeholders, such as health care professionals, educators and policy-makers, future problems could be prevented.

## Supplementary Information

**Additional file 1.**

**Additional file 2.**

**Additional file 3.**

## Data Availability

Data can be shared upon request with Deniz Harputlu (Deniz.Harputlu@ankara.edu.tr) of Jeroen Dikken (J.Dikken@hhs.nl).

## Abbreviations

KOPQ: Knowledge about Older Patients Quiz
KOPQ-US: Knowledge about Older Patients Quiz - United States of America
KOPQ-TR: Knowledge about Older Patients Quiz – Turkish version
S-CVI: Scale Content Validity Index
I-CVI: Item Content Validity Index
PSI: Person separation index
ISI: Item separation index
