# Supplementary material for: Hospital nurses’ knowledge about older patients in Turkey: a validation and comparison study
Source: BMC Nurs. 2022 May 10;21:111. doi: 10.1186/s12912-022-00882-6 (PMC9088085; doi:10.1186/s12912-022-00882-6)
Supplement: Supplementary file 2 — Additional file 2. [file 12912_2022_882_MOESM2_ESM.docx]

| **Appendix 2. Questions of the knowledge about Older Patient Quiz and percentage of correct answers of Dutch and Turkish nurses who answered the question correct.** | | | |
| --- | --- | --- | --- |
| Item nr. | Question of the Knowledge about Older Patient - Quiz | Percentage correct Dutch Nurses | Percentage correct Turkish nurses |
| 1 | Forgetfulness, concentration issues, and indecisiveness are parts of aging rather than indicators of depression. | 53.2% | 31,1% |
| 2 | Unexpected urinary incontinence in an older person may indicate that the person is suffering from a urinary tract infection. | 90.7% | 59,3% |
| 3 | Patients with a cognitive disorder, such as dementia, are at greater risk for delirium. | 94.8% | 87,4% |
| 4 | Malnutrition can have negative effects on thinking and observation skills. | 98.9% | 95,6% |
| 5 | In general, older people are more sensitive to medication because their kidney and liver functions are declining. | 86.1% | 92,6% |
| 6 | Meeting with families during patient assessment is required only for persons suffering from dementia. | 91.5% | 89,6% |
| 7 | For older people, bed rest is important to enhance recovery. | 97.2% | 44,4% |
| 8 | Patients rarely remember that they were anxious and/or restless during delirium. | 67.8% | 19,3% |
| 9 | Older people need less fluid because they exercise less. | 97.7% | 77,8% |
| 10 | Asking patients whether they have fallen in the past 6 months is a good way of assessing risk of falling. | 90.1% | 64,4% |
| 11 | Pressure that cuts off the blood supply to tissue for two hours may result in pressure ulcers. | 94.3% | 94,1% |
| 12 | Depression is recognized in older people less frequently than it is in younger people. | 96.9% | 74,8% |
| 13 | Lowering the frequency of a medication is an effective intervention to achieve (medication) adherence by patients. | 79.8% | 37,8% |
| 14 | Incontinent patients must have their soiled clothing changed but do not need to be placed on the toilet afterwards. | 98.4% | 97% |
| 15 | It is good to have older people drink more often, because they have a reduced thirst sensation. | 94.3% | 87,4% |
| 16 | In the case of delirium, bright lighting should be used to illuminate all of the corners of the room. | 70.3% | 79,3% |
| 17 | Medication may cause geriatric problems such as memory deficits, incontinence, falling, and depression. | 94.3% | 75,6% |
| 18 | Overburdening of family caregivers may lead to abuse of the person for whom they are providing care. | 94.8% | 84,4% |
| 19 | It is good to provide extensive instruction about how to complete tasks to patients with apraxia. | 58.2% | 15,6% |
| 20 | When speaking to hearing-impaired older patients, it is best to speak at normal volume. | 56.7% | 18,5% |
| 21 | An older person with a BMI of >25 cannot be undernourished. | 95.4% | 54,8% |
| 22 | In the case of difficulty swallowing, all medicines must be ground to ensure that patients ingest them. | 72.2% | 36,3% |
| 23 | In the case of depression, memory problems may occur. | 96.3% | 91,1% |
| 24 | Most family caregivers do not need additional support from homecare services. | 91.5% | 88,1% |
| 25 | As a nurse, you have to speak clearly into the ear of hearing-impaired older patients. | 61.7% | 9,6% |
| 26 | Pain medication should be administered to older people as little as possible, due to the possibility of addiction. | 86.9% | 31,1% |
| 27 | We identify pressure ulcers only if blister formation or abrasions have occurred. | 93.7% | 97% |
| 28 | In the case of delirium, activities should be spread out evenly over the day. | 98.5% | 81,5% |
| 29 | The risk of falling is higher for people in the hospital setting than in those who are living at home. | 65.3% | 56,3% |
| 30 | Stress incontinence may occur in patients who are not capable of opening their own trousers. | 62.9% | 8,9% |
| *40% difference between correct answers is bold | | | |
