# Supplementary material for: Hospital nurses’ knowledge about older patients in Turkey: a validation and comparison study
Source: BMC Nurs. 2022 May 10;21:111. doi: 10.1186/s12912-022-00882-6 (PMC9088085; doi:10.1186/s12912-022-00882-6)
Supplement: Supplementary file 3 — Additional file 3. [file 12912_2022_882_MOESM3_ESM.docx]

**Knowledge about Older Patients Quiz (KOP-Q) for Nurses**

For each statement, please answer “True” or “False”. Along the certainty bar, please indicate how certain you are about your answer (ranging from 0 – 100% certain).

|  |  | **True** | **False** |
| --- | --- | --- | --- |
| **1** | **Forgetfulness, concentration issues, and indecisiveness are parts of aging rather than indicators of depression.** | □ | □ |
| \| How certain are you about this answer? \| \| \| \| \| \| \| \| \| \|  \| \| --- \| --- \| --- \| --- \| --- \| --- \| --- \| --- \| --- \| --- \| --- \| \|  \|  \|  \|  \|  \|  \|  \|  \|  \|  \|  \| \|  \|  \|  \|  \|  \|  \|  \|  \|  \|  \|  \| \|  \|  \|  \|  \|  \|  \|  \|  \|  \|  \|  \| \| 0% \|  \|  \|  \|  \| 50% \|  \|  \|  \|  \| 100% \| | | | |
|  |  | **True** | **False** |
| **2** | **Unexpected urinary incontinence in an older person may indicate that the person is suffering from a urinary tract infection.** | □ | □ |
| \| How certain are you about this answer? \| \| \| \| \| \| \| \| \| \|  \| \| --- \| --- \| --- \| --- \| --- \| --- \| --- \| --- \| --- \| --- \| --- \| \|  \|  \|  \|  \|  \|  \|  \|  \|  \|  \|  \| \|  \|  \|  \|  \|  \|  \|  \|  \|  \|  \|  \| \|  \|  \|  \|  \|  \|  \|  \|  \|  \|  \|  \| \| 0% \|  \|  \|  \|  \| 50% \|  \|  \|  \|  \| 100% \| | | | |
|  |  | **True** | **False** |
| **3** | **Patientswith a cognitive disorder, such as dementia,are at greater risk for delirium.** | □ | □ |
| \| How certain are you about this answer? \| \| \| \| \| \| \| \| \| \|  \| \| --- \| --- \| --- \| --- \| --- \| --- \| --- \| --- \| --- \| --- \| --- \| \|  \|  \|  \|  \|  \|  \|  \|  \|  \|  \|  \| \|  \|  \|  \|  \|  \|  \|  \|  \|  \|  \|  \| \|  \|  \|  \|  \|  \|  \|  \|  \|  \|  \|  \| \| 0% \|  \|  \|  \|  \| 50% \|  \|  \|  \|  \| 100% \| | | | |
|  |  | **True** | **False** |
| **4** | **Malnutrition can have negative effectsonthinking and observation skills.** | □ | □ |
| \| How certain are you about this answer? \| \| \| \| \| \| \| \| \| \|  \| \| --- \| --- \| --- \| --- \| --- \| --- \| --- \| --- \| --- \| --- \| --- \| \|  \|  \|  \|  \|  \|  \|  \|  \|  \|  \|  \| \|  \|  \|  \|  \|  \|  \|  \|  \|  \|  \|  \| \|  \|  \|  \|  \|  \|  \|  \|  \|  \|  \|  \| \| 0% \|  \|  \|  \|  \| 50% \|  \|  \|  \|  \| 100% \| | | | |
|  |  | **True** | **False** |
| **5** | **In general,older people are moresensitive to medication becausetheir kidney and liver functions are declining.** | □ | □ |
| \| How certain are you about this answer? \| \| \| \| \| \| \| \| \| \|  \| \| --- \| --- \| --- \| --- \| --- \| --- \| --- \| --- \| --- \| --- \| --- \| \|  \|  \|  \|  \|  \|  \|  \|  \|  \|  \|  \| \|  \|  \|  \|  \|  \|  \|  \|  \|  \|  \|  \| \|  \|  \|  \|  \|  \|  \|  \|  \|  \|  \|  \| \| 0% \|  \|  \|  \|  \| 50% \|  \|  \|  \|  \| 100% \| | | | |
|  |  | **True** | **False** |
| **6** | **Meeting with families during patient assessment is required only for persons suffering from dementia.** | □ | □ |
| \| How certain are you about this answer? \| \| \| \| \| \| \| \| \| \|  \| \| --- \| --- \| --- \| --- \| --- \| --- \| --- \| --- \| --- \| --- \| --- \| \|  \|  \|  \|  \|  \|  \|  \|  \|  \|  \|  \| \|  \|  \|  \|  \|  \|  \|  \|  \|  \|  \|  \| \|  \|  \|  \|  \|  \|  \|  \|  \|  \|  \|  \| \| 0% \|  \|  \|  \|  \| 50% \|  \|  \|  \|  \| 100% \| | | | |

|  |  | **True** | **False** |
| --- | --- | --- | --- |
| **7** | **For older people,bed rest is important to enhance recovery.** | □ | □ |
| \| How certain are you about this answer? \| \| \| \| \| \| \| \| \| \|  \| \| --- \| --- \| --- \| --- \| --- \| --- \| --- \| --- \| --- \| --- \| --- \| \|  \|  \|  \|  \|  \|  \|  \|  \|  \|  \|  \| \|  \|  \|  \|  \|  \|  \|  \|  \|  \|  \|  \| \|  \|  \|  \|  \|  \|  \|  \|  \|  \|  \|  \| \| 0% \|  \|  \|  \|  \| 50% \|  \|  \|  \|  \| 100% \| | | | |
|  |  | **True** | **False** |
| **8** | **Patients rarely remember that they were anxious and/or restless during delirium.** | □ | □ |
| \| How certain are you about this answer? \| \| \| \| \| \| \| \| \| \|  \| \| --- \| --- \| --- \| --- \| --- \| --- \| --- \| --- \| --- \| --- \| --- \| \|  \|  \|  \|  \|  \|  \|  \|  \|  \|  \|  \| \|  \|  \|  \|  \|  \|  \|  \|  \|  \|  \|  \| \|  \|  \|  \|  \|  \|  \|  \|  \|  \|  \|  \| \| 0% \|  \|  \|  \|  \| 50% \|  \|  \|  \|  \| 100% \| | | | |
|  |  | **True** | **False** |
| **9** | **Older people need less fluid because they exercise less.** | □ | □ |
| \| How certain are you about this answer? \| \| \| \| \| \| \| \| \| \|  \| \| --- \| --- \| --- \| --- \| --- \| --- \| --- \| --- \| --- \| --- \| --- \| \|  \|  \|  \|  \|  \|  \|  \|  \|  \|  \|  \| \|  \|  \|  \|  \|  \|  \|  \|  \|  \|  \|  \| \|  \|  \|  \|  \|  \|  \|  \|  \|  \|  \|  \| \| 0% \|  \|  \|  \|  \| 50% \|  \|  \|  \|  \| 100% \| | | | |
|  |  | **True** | **False** |
| **10** | **Asking patients whetherthey have fallen in the past 6 months is a good way ofassessingrisk of falling.** | □ | □ |
| \| How certain are you about this answer? \| \| \| \| \| \| \| \| \| \|  \| \| --- \| --- \| --- \| --- \| --- \| --- \| --- \| --- \| --- \| --- \| --- \| \|  \|  \|  \|  \|  \|  \|  \|  \|  \|  \|  \| \|  \|  \|  \|  \|  \|  \|  \|  \|  \|  \|  \| \|  \|  \|  \|  \|  \|  \|  \|  \|  \|  \|  \| \| 0% \|  \|  \|  \|  \| 50% \|  \|  \|  \|  \| 100% \| | | | |
|  |  | **True** | **False** |
| **11** | **Pressure that cuts offthe blood supply to tissuefor two hours may result in pressure ulcers.** | □ | □ |
| \| How certain are you about this answer? \| \| \| \| \| \| \| \| \| \|  \| \| --- \| --- \| --- \| --- \| --- \| --- \| --- \| --- \| --- \| --- \| --- \| \|  \|  \|  \|  \|  \|  \|  \|  \|  \|  \|  \| \|  \|  \|  \|  \|  \|  \|  \|  \|  \|  \|  \| \|  \|  \|  \|  \|  \|  \|  \|  \|  \|  \|  \| \| 0% \|  \|  \|  \|  \| 50% \|  \|  \|  \|  \| 100% \| | | | |
|  |  | **True** | **False** |
| **12** | **Depression is recognized in older people less frequently than it is inyounger people.** | □ | □ |
| \| How certain are you about this answer? \| \| \| \| \| \| \| \| \| \|  \| \| --- \| --- \| --- \| --- \| --- \| --- \| --- \| --- \| --- \| --- \| --- \| \|  \|  \|  \|  \|  \|  \|  \|  \|  \|  \|  \| \|  \|  \|  \|  \|  \|  \|  \|  \|  \|  \|  \| \|  \|  \|  \|  \|  \|  \|  \|  \|  \|  \|  \| \| 0% \|  \|  \|  \|  \| 50% \|  \|  \|  \|  \| 100% \| | | | |
|  |  | **True** | **False** |
| **13** | **Lowering the frequency of a medication is an effective intervention to achieve (medication) adherenceby patients.** | □ | □ |
| \| How certain are you about this answer? \| \| \| \| \| \| \| \| \| \|  \| \| --- \| --- \| --- \| --- \| --- \| --- \| --- \| --- \| --- \| --- \| --- \| \|  \|  \|  \|  \|  \|  \|  \|  \|  \|  \|  \| \|  \|  \|  \|  \|  \|  \|  \|  \|  \|  \|  \| \|  \|  \|  \|  \|  \|  \|  \|  \|  \|  \|  \| \| 0% \|  \|  \|  \|  \| 50% \|  \|  \|  \|  \| 100% \| | | | |

|  |  | **True** | **False** |
| --- | --- | --- | --- |
| **14** | **Incontinent patientsmust have their soiled clothing changed but donot need to be placed on the toiletafterwards.** | □ | □ |
| \| How certain are you about this answer? \| \| \| \| \| \| \| \| \| \|  \| \| --- \| --- \| --- \| --- \| --- \| --- \| --- \| --- \| --- \| --- \| --- \| \|  \|  \|  \|  \|  \|  \|  \|  \|  \|  \|  \| \|  \|  \|  \|  \|  \|  \|  \|  \|  \|  \|  \| \|  \|  \|  \|  \|  \|  \|  \|  \|  \|  \|  \| \| 0% \|  \|  \|  \|  \| 50% \|  \|  \|  \|  \| 100% \| | | | |
|  |  | **True** | **False** |
| **15** | **It is good to have older people drink more often, because they have a reduced thirst sensation.** | □ | □ |
| \| How certain are you about this answer? \| \| \| \| \| \| \| \| \| \|  \| \| --- \| --- \| --- \| --- \| --- \| --- \| --- \| --- \| --- \| --- \| --- \| \|  \|  \|  \|  \|  \|  \|  \|  \|  \|  \|  \| \|  \|  \|  \|  \|  \|  \|  \|  \|  \|  \|  \| \|  \|  \|  \|  \|  \|  \|  \|  \|  \|  \|  \| \| 0% \|  \|  \|  \|  \| 50% \|  \|  \|  \|  \| 100% \| | | | |
|  |  | **True** | **False** |
| **16** | **In the case of delirium, bright lighting should be used to illuminate all of the corners of the room.** | □ | □ |
| \| How certain are you about this answer? \| \| \| \| \| \| \| \| \| \|  \| \| --- \| --- \| --- \| --- \| --- \| --- \| --- \| --- \| --- \| --- \| --- \| \|  \|  \|  \|  \|  \|  \|  \|  \|  \|  \|  \| \|  \|  \|  \|  \|  \|  \|  \|  \|  \|  \|  \| \|  \|  \|  \|  \|  \|  \|  \|  \|  \|  \|  \| \| 0% \|  \|  \|  \|  \| 50% \|  \|  \|  \|  \| 100% \| | | | |
|  |  | **True** | **False** |
| **17** | **Medicationmay cause geriatricproblemssuch as memory deficits, incontinence, falling,and depression.** | □ | □ |
| \| How certain are you about this answer? \| \| \| \| \| \| \| \| \| \|  \| \| --- \| --- \| --- \| --- \| --- \| --- \| --- \| --- \| --- \| --- \| --- \| \|  \|  \|  \|  \|  \|  \|  \|  \|  \|  \|  \| \|  \|  \|  \|  \|  \|  \|  \|  \|  \|  \|  \| \|  \|  \|  \|  \|  \|  \|  \|  \|  \|  \|  \| \| 0% \|  \|  \|  \|  \| 50% \|  \|  \|  \|  \| 100% \| | | | |
|  |  | **True** | **False** |
| **18** | **Overburdening of family caregivers may lead to abuse of the person for whom they are providing care.** | □ | □ |
| \| How certain are you about this answer? \| \| \| \| \| \| \| \| \| \|  \| \| --- \| --- \| --- \| --- \| --- \| --- \| --- \| --- \| --- \| --- \| --- \| \|  \|  \|  \|  \|  \|  \|  \|  \|  \|  \|  \| \|  \|  \|  \|  \|  \|  \|  \|  \|  \|  \|  \| \|  \|  \|  \|  \|  \|  \|  \|  \|  \|  \|  \| \| 0% \|  \|  \|  \|  \| 50% \|  \|  \|  \|  \| 100% \| | | | |
|  |  | **True** | **False** |
| **19** | **It is good to provide extensiveinstruction about how to complete tasks to patients with apraxia.** | □ | □ |
| \| How certain are you about this answer? \| \| \| \| \| \| \| \| \| \|  \| \| --- \| --- \| --- \| --- \| --- \| --- \| --- \| --- \| --- \| --- \| --- \| \|  \|  \|  \|  \|  \|  \|  \|  \|  \|  \|  \| \|  \|  \|  \|  \|  \|  \|  \|  \|  \|  \|  \| \|  \|  \|  \|  \|  \|  \|  \|  \|  \|  \|  \| \| 0% \|  \|  \|  \|  \| 50% \|  \|  \|  \|  \| 100% \| | | | |
|  |  | **True** | **False** |
| **20** | **When speaking tohearing-impairedolder patients, it is best to speak at normal volume.** | □ | □ |
| \| How certain are you about this answer? \| \| \| \| \| \| \| \| \| \|  \| \| --- \| --- \| --- \| --- \| --- \| --- \| --- \| --- \| --- \| --- \| --- \| \|  \|  \|  \|  \|  \|  \|  \|  \|  \|  \|  \| \|  \|  \|  \|  \|  \|  \|  \|  \|  \|  \|  \| \|  \|  \|  \|  \|  \|  \|  \|  \|  \|  \|  \| \| 0% \|  \|  \|  \|  \| 50% \|  \|  \|  \|  \| 100% \| | | | |

|  |  | **True** | **False** |
| --- | --- | --- | --- |
| **21** | **An older person with a BMI of >25 cannot be undernourished.** | □ | □ |
| \| How certain are you about this answer? \| \| \| \| \| \| \| \| \| \|  \| \| --- \| --- \| --- \| --- \| --- \| --- \| --- \| --- \| --- \| --- \| --- \| \|  \|  \|  \|  \|  \|  \|  \|  \|  \|  \|  \| \|  \|  \|  \|  \|  \|  \|  \|  \|  \|  \|  \| \|  \|  \|  \|  \|  \|  \|  \|  \|  \|  \|  \| \| 0% \|  \|  \|  \|  \| 50% \|  \|  \|  \|  \| 100% \| | | | |
|  |  | **True** | **False** |
| **22** | **In the case of difficulty swallowing,all medicines must be ground to ensure that patientsingest them.** | □ | □ |
| \| How certain are you about this answer? \| \| \| \| \| \| \| \| \| \|  \| \| --- \| --- \| --- \| --- \| --- \| --- \| --- \| --- \| --- \| --- \| --- \| \|  \|  \|  \|  \|  \|  \|  \|  \|  \|  \|  \| \|  \|  \|  \|  \|  \|  \|  \|  \|  \|  \|  \| \|  \|  \|  \|  \|  \|  \|  \|  \|  \|  \|  \| \| 0% \|  \|  \|  \|  \| 50% \|  \|  \|  \|  \| 100% \| | | | |
|  |  | **True** | **False** |
| **23** | **In the case of depression,memory problemsmay occur.** | □ | □ |
| \| How certain are you about this answer? \| \| \| \| \| \| \| \| \| \|  \| \| --- \| --- \| --- \| --- \| --- \| --- \| --- \| --- \| --- \| --- \| --- \| \|  \|  \|  \|  \|  \|  \|  \|  \|  \|  \|  \| \|  \|  \|  \|  \|  \|  \|  \|  \|  \|  \|  \| \|  \|  \|  \|  \|  \|  \|  \|  \|  \|  \|  \| \| 0% \|  \|  \|  \|  \| 50% \|  \|  \|  \|  \| 100% \| | | | |
|  |  | **True** | **False** |
| **24** | **Mostfamily caregivers donot need additional support from homecare services.** | □ | □ |
| \| How certain are you about this answer? \| \| \| \| \| \| \| \| \| \|  \| \| --- \| --- \| --- \| --- \| --- \| --- \| --- \| --- \| --- \| --- \| --- \| \|  \|  \|  \|  \|  \|  \|  \|  \|  \|  \|  \| \|  \|  \|  \|  \|  \|  \|  \|  \|  \|  \|  \| \|  \|  \|  \|  \|  \|  \|  \|  \|  \|  \|  \| \| 0% \|  \|  \|  \|  \| 50% \|  \|  \|  \|  \| 100% \| | | | |
|  |  | **True** | **False** |
| **25** | **As a nurse, you have to speak clearly into the ear of hearing-impairedolder patients.** | □ | □ |
| \| How certain are you about this answer? \| \| \| \| \| \| \| \| \| \|  \| \| --- \| --- \| --- \| --- \| --- \| --- \| --- \| --- \| --- \| --- \| --- \| \|  \|  \|  \|  \|  \|  \|  \|  \|  \|  \|  \| \|  \|  \|  \|  \|  \|  \|  \|  \|  \|  \|  \| \|  \|  \|  \|  \|  \|  \|  \|  \|  \|  \|  \| \| 0% \|  \|  \|  \|  \| 50% \|  \|  \|  \|  \| 100% \| | | | |
|  |  | **True** | **False** |
| **26** | **Pain medication should be administered to older people as little as possible, due to the possibility of addiction.** | □ | □ |
| \| How certain are you about this answer? \| \| \| \| \| \| \| \| \| \|  \| \| --- \| --- \| --- \| --- \| --- \| --- \| --- \| --- \| --- \| --- \| --- \| \|  \|  \|  \|  \|  \|  \|  \|  \|  \|  \|  \| \|  \|  \|  \|  \|  \|  \|  \|  \|  \|  \|  \| \|  \|  \|  \|  \|  \|  \|  \|  \|  \|  \|  \| \| 0% \|  \|  \|  \|  \| 50% \|  \|  \|  \|  \| 100% \| | | | |
|  |  | **True** | **False** |
| **27** | **We identify pressure ulcersonly ifblister formation or abrasions have occurred.** | □ | □ |
| \| How certain are you about this answer? \| \| \| \| \| \| \| \| \| \|  \| \| --- \| --- \| --- \| --- \| --- \| --- \| --- \| --- \| --- \| --- \| --- \| \|  \|  \|  \|  \|  \|  \|  \|  \|  \|  \|  \| \|  \|  \|  \|  \|  \|  \|  \|  \|  \|  \|  \| \|  \|  \|  \|  \|  \|  \|  \|  \|  \|  \|  \| \| 0% \|  \|  \|  \|  \| 50% \|  \|  \|  \|  \| 100% \| | | | |

|  |  | **True** | **False** |
| --- | --- | --- | --- |
| **28** | **In the case of delirium, activities should be spread out evenly over the day.** | □ | □ |
| \| How certain are you about this answer? \| \| \| \| \| \| \| \| \| \|  \| \| --- \| --- \| --- \| --- \| --- \| --- \| --- \| --- \| --- \| --- \| --- \| \|  \|  \|  \|  \|  \|  \|  \|  \|  \|  \|  \| \|  \|  \|  \|  \|  \|  \|  \|  \|  \|  \|  \| \|  \|  \|  \|  \|  \|  \|  \|  \|  \|  \|  \| \| 0% \|  \|  \|  \|  \| 50% \|  \|  \|  \|  \| 100% \| | | | |
|  |  | **True** | **False** |
| **29** | **The risk of falling is higher for people in the hospital setting than in those who are living at home.** | □ | □ |
| \| How certain are you about this answer? \| \| \| \| \| \| \| \| \| \|  \| \| --- \| --- \| --- \| --- \| --- \| --- \| --- \| --- \| --- \| --- \| --- \| \|  \|  \|  \|  \|  \|  \|  \|  \|  \|  \|  \| \|  \|  \|  \|  \|  \|  \|  \|  \|  \|  \|  \| \|  \|  \|  \|  \|  \|  \|  \|  \|  \|  \|  \| \| 0% \|  \|  \|  \|  \| 50% \|  \|  \|  \|  \| 100% \| | | | |
|  |  | **True** | **False** |
| **30** | **Stress incontinencemay occur in patients who are not capable of opening their own trousers.** | □ | □ |
| \| How certain are you about this answer? \| \| \| \| \| \| \| \| \| \|  \| \| --- \| --- \| --- \| --- \| --- \| --- \| --- \| --- \| --- \| --- \| --- \| \|  \|  \|  \|  \|  \|  \|  \|  \|  \|  \|  \| \|  \|  \|  \|  \|  \|  \|  \|  \|  \|  \|  \| \|  \|  \|  \|  \|  \|  \|  \|  \|  \|  \|  \| \| 0% \|  \|  \|  \|  \| 50% \|  \|  \|  \|  \| 100% \| | | | |

**Answer Key KOP-Q (30 item)**

Every correct answer on the knowledge questionnaire receives 1 point, and every incorrect answer receives 0 points (total score: minimum = 0, maximum = 30). The average of the certainty scores can be calculated by summing all of the percentages provided per question divided by 30.

| 1 | FALSE | 11 | TRUE | 21 | FALSE |
| --- | --- | --- | --- | --- | --- |
| 2 | TRUE | 12 | TRUE | 22 | FALSE |
| 3 | TRUE | 13 | TRUE | 23 | TRUE |
| 4 | TRUE | 14 | FALSE | 24 | FALSE |
| 5 | TRUE | 15 | TRUE | 25 | FALSE |
| 6 | FALSE | 16 | FALSE | 26 | FALSE |
| 7 | FALSE | 17 | TRUE | 27 | FALSE |
| 8 | FALSE | 18 | TRUE | 28 | TRUE |
| 9 | FALSE | 19 | FALSE | 29 | TRUE |
| 10 | TRUE | 20 | TRUE | 30 | FALSE |
